# Etiology of Caries

**Published:** 1889-08

**Authors:** W. C. Wardlaw


ARTICLE II.

### ETIOLOGY OF CARIES.

W. C. WARDLAW, M. D., D. D. S.

(Read before the Georgia State Dental Society, June 12, 1889).

"Dr., why should my teeth decay so? My mother's did not." "Dr., why does not sister have the toothache? She does not take half the care of her teeth that I do of mine." "Dr., I send my daughter to the dentist regularly, and I have her to brush her teeth well; yet, she always requires some of them to be refilled. I do not understand it?"

Gentlemen, have you not had such unwelcome questions hurled at you? Are you prepared to answer them satisfactorily to yourself and patient? Do you have to "whip the devil around the stump" with indefinite generalities, or have to cover your retreat with a flow of technical terms?

It is not begging the question to say, " It is because of your fashionable, artificial mode of life; it is because of your environments; it is because you eat slops and soft-boiled food; it is because of the modern degeneracy of the human race; it is because you do not efficiently clean your teeth." Whilst there may be a modicum of truth in all these answers, they scarcely meet the case, for these are only predisposing causes, and the last one alone, a possible explanation.

No disease is so universally prevalent as "caries." All nations, ages and sexes are subject to it. If you will observe your own families, I think that you will find that the services of the dentist are needed ten times, where those of the physician are required once.

The cardinal principle of therapeutics is, remove the cause and the effect will cease: " *Tolle causam, effectus cessat.*" Hence, it behooves us as dental specialists, to discover the primary cause of "caries," that we may successfully combat or regulate them.

Let us then, as scientific men, look at the question with a view of satisfying our minds with an answer having all the fullness and exactness obtainable from the present state of acquired knowledge.

Nationality has a supposed predisposing influence, Americans being said to have the worst teeth; but I do not believe our statistics are sufficiently accurate to warrant definite conclusions upon this point. Heredity has its undoubted effects, particularly in determining the forms and arrangements of the teeth; and, in a more ill-defined way, of transmitting from parent to child the peculiar character of the cellular elements, rendering the child susceptible to disease. The influence of sex is positive; and, in the case of pregnant females, their excessive liability to caries is to be explained upon the principle of the vitiated condition of the fluids of the mouth, consequent upon this *systemic disorder*, so to speak; their corrosive properties being thus increased. To mal-formation of individual teeth, is directly traceable one of the causes predisposing to caries.

But we pass by all of these secondary causes, and come to study the theory of decay, its *modus operandi*, its philosophy.

From time to time since dentistry began, different theories of caries have been promulgated, and advocated with degrees of plausibility in proportion to the knowledge of the period; but of them we will merely take a passing glance, relegating them to the historic past, where they have been thrust by the exhaustive investigations of modern days.

Inflammation, at first ill-defined, but subsequently located in the lining membrane of the pulp chamber, was once assigned as an unexplainable cause of caries. This made decay to begin in the internal structure of the tooth, an error which, in the light of our present knowledge, seems wonderful of entertainment: yet, I myself have heard it maintained that caries could so begin, and the late edition of Harris says, "It usually commences in the outer surface of the dentine of the crown, *beneath* the enamel." Subsequent observers promulgated the theory, that the phenomena of caries were to be accounted for by some sort of chemical decomposition, but their ideas were crude and conflicting. The chemico-vital and the galvano-electric, with other vague theories, the light of investigation soon put to refutation.

One recent theory, however, the most plausible and most generally accepted, was that of Dr. Geo. Watt, of Xenia, Ohio, who made the mineral acids to do the work of destruction in the dental organs. The action of these acids upon the lime-salts of the teeth. But it did not fulfill all of the requirements of the case, and left some of the phenomena of caries unexplained. The principal difficulty in the way, was to supply the acids in sufficient quantities and intensity, at the spot of decay. Dr. Watt made labored and intelligent effort to explain the chemical action and reaction taking place in the mouth, necessary to furnish his sulphuric, hydro-chloric and nitric acids, in quantities, minute it is true, but in their nascent condition, of sufficient strength. He could not, however, produce upon teeth outside of the mouth, decay, having all the characteristics of true caries. It was also urged in opposition, that if mineral acids were in the mouth, in sufficient quantities to produce caries upon, say, the approximal surface of a tooth, they would necessarily produce a perceptible effect upon other surfaces.

This theory, supported by a number of scientific facts, has much in it to commend it to general favor, but it does not go far enough. It is correct, in that it makes caries the result of chemical action, but it does not supply the right acid, the lactic.

It has been supplanted by the more recent "germ" theory, to which form has been given principally by Miller, of Berlin. Some have attempeted to cast ridicule upon it, by calling it the "bug" theory, the "worm" theory, etc., but it has "come to stay," and to my mind fulfills all the requirements of a perfect theory, and explains the hitherto obscure phenomena of caries, more satisfactorily than anything which preceded it. It is, in brief, this: fermentation of organic matters, in contact with the teeth, is caused by bacteria, which generate lactic acid, by which the teeth are decomposed.

For a number of years, there have been vague notions entertained by thinkers, more advanced than their times, that in some mysterious way, corruption, fermentation, putrefaction, suppuration, disease, death, decomposition were due to the influence of living organisms, too minute to be observed by ordinary vision. The microscope has since substantiated these views, in a large measure, by establishing the fact that microbes, bacteria, fungi, micro-organisms, etc., are associated in innumerable multitudes, with these processes. These minute organic germs are present in the atmosphere, earth and water, and seem to be well-nigh universal.

The natural query then, suggested was, what is the specific part played by them in these metamorphoses?

Analogy, if not natural philosophy, requires that every living organism must have nutriment upon which to subsist, hence these microbes were supposed to derive their food supply by a destructive process or function, upon the matter with which they were in immediate contact.

Now, more than ever before, is this subject engaging the close study of the medical and scientific world, and is of importance sufficient to command our attention for this little while. To the pains-taking and laborious investigations of Lester, Koch, Pasteur and Miller, are we indebted for our present knowledge on the subject. They have proved, not merely the existence of these micrococci, and their association with abnormal conditions, but have demonstrated to the satisfaction of many minds, that to the various diseases there belong their specific micrococci; and have done this, not only by removing and excluding them, and thus arresting and preventing disease, but also by propagating particular diseases by inoculating the lower animals with their specific microbe. E. G.—A rat inoculated with cholera microbes, will have not smallpox, but cholera. Infection and decomposition therefore seemed to be their function.

Schwann discovered the yeast-plant to be the active agent in alcoholic fermentation. The action of this yeast-plant or ferment, is to decompose, or separate the grape sugar of the wine into alcohol and carbonic acid. It is a minute vegetable fungus, capable of propagating itself indefinitely in its proper medium. A small quantity, added to a culture medium, will soon set up fermentation, and a portion taken from this solution will continue the process in a second culture, and so on. This was then thought to be the type of the fermentation, or change, at the bottom of all of these decompositions. The itch, cholera, measles, eruptive fevers, etc., were thought to be propagated through infection by fermenting germs. What, then, is their mode af action? Obviously, the first consideration is to determine the nature of fermentation in general.

Organic matter, vegetable and animal, when subjected to the conditions of warmth and moisture, for a sufficient length of time, gradually begin to decompose, or putrefy, always having associated with the process some kind of micro-organisms, and always exhibiting an acid reaction. This is fermentation, and is, instead of death, a real physiological process of a living organism; digestion, assimilation and excretion, all physiological processes, are actually taking place in the *persons*, so to speak, of these little fungi.

If these little parasites, or fungi, be collected in quantity, dried and washed in distilled water, the solution, when evaporated, will be found to contain an inorganic ferment, or solvent, which is capable of dissolving or digesting, and preparing for assimilation by the organisn, its proper food material. Such is the ptyalin of the saliva, a ferment, solvent or disastase, which decomposes or breaks up the starch granules into sugar, the form necessary for assimilation. So is pepsin the active principle, or digestive solvent in the gastric fluid. The sugar converted, or prepared as explained, is in the ordinary function of digestion, appropriated or assimilated by the animal; in case of fermentation proper, the microbe further decomposes or separates it into alcohol and carbonic acid.

Fermentation proper, therefore, cannot take place without the aid of microbes, organic ferments. Our object now is to show that caries results from fermentation of food, mucous, saliva and organic matter about the teeth.

Dr. Miller being a practical dentist, as well as expert microscopist, devoted himself to the study of the bacteria of the mouth, with especial reference to their agency in caries. Microbes were found by the aid of the microscope, in both the saliva and carious dentine, in great numbers and in great varieties. Saliva was sterilized, that is, all of the germs in it were destroyed, and the further access of germs being prevented, no fermentation took place. Microbes were then introduced and fermentation began at once, and from this fermentation-culture a portion was taken and added to other sterilized saliva, and it fermented also. When an antiseptic was added, no fermentation ensued. Thus the process was continued, establishing the facts that nothing in pure, sterilized saliva would induce fermentation; that living organisms would cause it; that germs were essential to it; and that these microbes could be propagated indefinitely. Similar investigations, with similar results, were had as to carious dentine and its bacteria.

Having thus reasoned from cause to effect, he reasoned from effect to cause, by taking carious dentine and with it impregnated a culture solution, into which sound dentine was introduced, the result showing fermentation and the decomposition of the dentine. With a portion from this culture medium, the experiment was continued, and with similar results.

An important step, a very difficult and tedious one, was to ascertain the particular species of micrococci concerned in the fermentation of caries. To do this, as small a number as could be separated of germs, was taken from deep-seated caries and examined, and implanted in cultures, from which a particular variety would be selected, and gradually there was thus isolated a species which could always be found in caries, and which by itself alone was able to cause decay. An acid was always present with this uncrocyccus. To prove that this microbe could produce caries, sound dentine was placed first in a sterilized solution and there remained unaffected; but when the sound dentine was introduced into the solution to which the microbe had been added, decomposition ensued and this dissolution had all the characteristics of true caries.

Miller thus proved not only the presence of bacteria in caries, but the necessity of their presence. With them, artificial caries could be produced at will; without them it could not be at all.

If they be thus closely associated with caries, as its cause, how do they produce their effect? What is their mode of action? Wherever they were found, invariably an acid reaction could be detected, this being an essential characteristic of fermentation everywhere. Further investigation showed lactic acid to be the natural product of these microbes; that it was constantly present in caries; and that it is the element decomposing the lime-salts of the teeth.

This then, is the process. Particles of food, mucus, or saliva, wedged between the teeth, or hid away in some fissure, or lying in some depression, or adhering to some protected surface of a tooth, and not removed by friction, nor dissolved or washed away by the fluids of the mouth, is taken posession of by bacteria, and fermentation begins; lactic acid, the effete matter of the microbes is formed, and immediately unites with the lime of the enamel; a slight depression is here made, giving more secure lodgement for additional food, and further their fermentation continues; the enamel armor is thus gradually pierced, and the less dense dentine is exposed; ingress is had by the bacteria; they consume the organic elements of the dentine; lactic acid is secreted more abundantly, it presses foward in advance breaking down the walls of the tubuli; the micrococci follow closely on, crowding in and packing full the tubuli; a secure foothold having been obtained, destruction runs riot; softening and breaking down proceeds in all directions; and we have a typical case of "caries."

Bacteria cannot penetrate the enamel in the way that the sawyer-worm bores into the pine wood, and the idea that such was necessarily the case, presented one of the great obstacles in the way of an early acceptance of the "germ" theory. But a foot-hold, a base of operations, a "point de appui," as the military men would say, having been obtained for them, by the chemical action of the lactic acid, which they secrete, (or in any other way), the process goes on, slowly at first, but increasing in intensity and rapidity.

What, then, are the obvious teachings of this paper? First, that clêanliness, absolute freedom of the teeth from the contact of fermenting matter, will positively prevent decay.

Second. That fermentation must be arrested, or prevented with antiseptics.

Third. That bacteria must be destroyed with germicides.

Fourth. That receptacles for the lodgement of bacteria, cavities of decay, must be hermetically sealed with materials not subject to their influence, so as to prevent their entrance.

We also learn why it is that the adjoining approximal surfaces of the teeth are so constantly found to be similarly affected by caries. And I am now prepared to believe what I did not formerly, that a number of cavities of decay in a mouth are so many hot-beds for the propagation of innumerable micro-organisms, which communicate caries by infection to teeth, which might otherwise remain sound.

A common error in regard to caries is the belief that the acids, causing the decay, are introduced with the food and fluids into the mouth, whereas it should be born in mind that they are generated at the very locality where they "get in" their work. It matters little, therefore, as to caries whether the saliva be neutral, alkaline or acid, except in so far as its condition may modify the fermentation of organic matters actually taking place at the points of decay.

Example; I used to be much bothered by seeing teeth decay in mouths where there were large incrustations of tartar. Caries was, at that time, supposed to be due to the acid condition of the saliva, and yet here was decay taking taking place in the very presence of an alkali, which ought to have neutralized the acid. The inconsistency I could not explain.

Of course it is understood that organic acids, malic, citric, acetic and other acids, under favoring circumstances, will unite with the lime-salts of the teeth and thus cause a destructive wasting, but "caries" proper, as ordinarily seen in the teeth, is the result of the destructive action of bacteria.—*Southern Dental Journal.*

